# The Making of the Vindolanda Wooden Writing Tablets: A Noninvasive Multianalytical Protocol for the Characterisation of Black Roman Inks

**DOI:** 10.1155/jamc/5142007

**Published:** 2026-02-08

**Authors:** Giovanna Vasco, Joanne Dyer, Richard Hobbs, Caroline R. Cartwright

**Affiliations:** ^1^ Department of Scientific Research, British Museum, London, WC1B 3DG, UK, britishmuseum.org; ^2^ Department of Britain, Europe and Prehistory, British Museum, London, WC1B 3DG, UK, britishmuseum.org

**Keywords:** black ink, carbon black, Raman spectroscopy, Vindolanda tablets

## Abstract

Within the ‘Making History’ project, the British Museum investigated the materiality of the Vindolanda ink writing tablets for the first time, with a particular focus on the possible differentiation of the ink sources employed. Thanks to the application of complementary scientific techniques, it was possible to develop an analytical protocol for the documentation of the ink writing from the palaeographic and conservation points of view and the characterisation of their manufacture. From the macroscopic to the microscopic scale, the use of imaging techniques (MBI, SWIR and digital microscope) highlighted the unique features of the different handwriting styles and allowed the identification of the ink typology. Raman spectroscopy and elemental analyses (XRF and SEM‐EDX) were then applied to gain information about the chemical–physical nature of the inks. The main achievement was the possibility to differentiate the sources of carbon‐based inks using Raman spectroscopy and multivariate analysis. It was possible to examine ink production at the edge of the Roman Empire, comparing it with the ancient literature and the different practices arising in the Mediterranean area.

## 1. Introduction

Within the development of written communication across different civilisations, black writing ink has a varied and incompletely explored history. In written sources, carbon‐ and plant‐based media and iron‐gall inks have been attested as comprising three main types, established in different periods, possibly due to economic factors and changes in the writing substrate [1]. Carbon‐based compounds were obtained from burnt organic and inorganic materials (i.e. wood, oil and earth) to obtain soots and charcoals [2]. They were stored as dry cakes to be mixed with a binder (gum arabic or animal glue) and suspended in a water‐soluble medium when required. Plant inks were brownish dyes resulting from aqueous solutions based on tannins with a possible binder [2, 3]. Iron‐gall inks are metal‐based compounds, containing a variable amount of manganese, copper, iron, lead or zinc [4–7]. Typical production methods involve the reaction between metallic sulphates, called vitriols, and gallic or tannic acid from oak gall and the subsequent oxidation of the developed complexes [4–7]. Over the past few years, further studies have proven the complexity of the iron‐gall recipes and the diversity of their formulations [8–11]. Among the different categories of ink, the oldest known ink is the carbon‐based variety, which was predominantly used until the development of iron‐gall ink in Egypt or Palestine in the third century, and its spread in Europe, becoming standard practice from the Middle Ages until the twentieth century [2, 3, 12].

Despite the extensive use of black inks through time and different geographical areas, there is a lack of information about their manufacture and the transition from one typology to another [2]. Only a few ancient recipes have been passed down from antiquity, especially in the Mediterranean area, in contrast with the variety of known treatises in the Middle East during Late Antiquity, Greek magical papyri (third and fourth century), and from the Middle Ages [1, 13–16]. The main literary sources in antiquity were written in the first century CE by Vitruvius, Pliny the Elder and Dioscorides [2, 17–19]. The term *mélav* or *atramentum*, which is used for black, was used as a synonym of ink to describe various black substances and not to refer to specific types [2]. The shift in use to different forms of ink involved a transition phase which is still unclear, with the use of heterogeneous mixtures of carbon black and metal‐bearing minerals [3, 20, 21]. Indeed, metal traces have been found in ancient carbon‐based inks derived from soot collected from copper or bronze vessels or from the byproducts of the manufacture of glass or metal smithing [2, 20, 22]. The intentional use of metal compounds over vast distances of space and time remains unknown [16, 18]. The application of chemical–physical methods of investigation allows some light to be shed on ink production where historical sources are insufficient [3, 22–25]. As a result of such ink characterisation efforts, a shift from the use of pure carbon seems to have already occurred in the Hellenistic period, as found in some scrolls from Herculaneum [3, 23, 24, 26–29]. Despite the spread of metal‐bearing inks, mixed inks also survived in later contexts, as observed in Coptic fragments dated between the seventh and eighth centuries in Bawit [30, 31].

Extensive scientific studies have been carried out to increase the legibility of ancient texts and to understand the severe degradation of iron‐gall inks [5, 24, 32], but the specific identification of black inks is still challenging. Establishing a multianalytical approach is required to exclude the misunderstanding of metal impurities and specifically identify the carbon‐based materials in compounds generically classified only as ‘carbon black’ [20, 33–37]. Due to the necessary preservation of the content of texts, noninvasive and nondestructive analytical techniques are preferable [25, 38].

Imaging‐based methods, such as multispectral and multiband imaging (MSI and MBI), and short‐wave infrared (SWIR), have been used to study the different categories of ink by exploiting their varied optical properties in the infrared (IR) range [38–40]. Iron‐gall inks lose their opacity in the near‐infrared (NIR) region, becoming invisible beyond 1400 nm, while plant‐based inks gradually fade, reaching transparency at 750 nm [40]. Carbon black, however, maintains its opacity throughout the IR region. Microscopic images can additionally help the identification by enabling clear visualisations of the penetration of the media into the substrate and the morphology of the ink particles. Inks that appear only on the surface of the substrate are characteristic of carbon black, while, in cases where considerable penetration into the writing substrate is observed, it is possible to identify either plant inks, due to their homogeneous appearance, and/or iron‐gall inks due to the nonuniform distribution of dark crystals. X‐ray techniques are suitable for the characterisation and quantification of metallic content in relation to an unintentional or deliberate addition of metals or the use of several vitriols. X‐ray phase contrast tomography (XPCT), computed tomography (CT) and magnetic resonance imaging (MRI) have also been used to distinguish the writing from the carbonised Herculaneum scrolls thanks to the metals used as a contrast agent [27]. An in‐depth investigation of the sources of carbon black is still challenging, but in the last decade, the use of Raman spectroscopy has gained popularity due to its ability to distinguish between several precursors and sources, although its application has yet to be considered the norm [25, 41–46].

At the British Museum (BM), part of an interdisciplinary project on hundreds of Roman writing tablets excavated at the Roman fort of Vindolanda aimed to study their manufacture. In this paper, the assessment is described as a noninvasive standard protocol on a first selection of tablets for the characterisation of carbon‐based inks. It was thus possible to maximise the classification of the inks, looking for possible relationships with their contents and giving a glimpse into their manufacture in Roman society.

## 2. Vindolanda

### 2.1. The Vindolanda Fort

**FIGURE 1:**
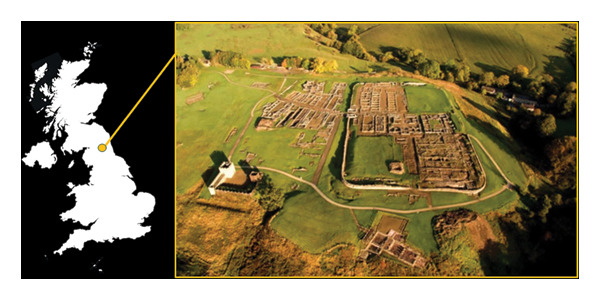
The fort and garrison settlement at Vindolanda (Image ©The Vindolanda Trust).

The Roman fort of Vindolanda is one of the most significant archaeological sites in Britain, offering a detailed picture of life on the northern frontier of the Roman Empire [47–49]. Located just south of Hadrian’s Wall in Northumberland, England, Vindolanda served as a key military and civilian settlement. The site of Vindolanda was initially occupied around AD 85, as part of a network of defences designed to control the local population and protect the Roman province of Britannia from the unconquered peoples to the north. The fort was rebuilt several times over the centuries, first in turf and timber and later in stone, reflecting changes in Roman military strategy and following the evolution of the garrison, close to Hadrian’s Wall. Then, after the Romans withdrew from Britain in the early fifth century, Vindolanda was gradually abandoned and rediscovered only in the nineteenth century. Nowadays, it is a major archaeological site and museum (Figure 1), managed by the Vindolanda Trust (VT).

The archaeological site is remarkable not only for having reflected the daily life of the Roman auxiliaries on the frontier with numerous uncovered artefacts, including pottery, weapons, glass, tools and jewellery, but especially for the exceptional preservation of organic items, such as clothing, wooden objects and structures, and the largest collection of leather shoes and writing tablets from any site in the Roman Empire. From these finds, it has been possible to gain an invaluable insight into the social, administrative and economic aspects of life at the front, highlighting the presence of a complete community, with entire families including women and children and with other civilians such as traders and artisans, thus allowing the reconstruction of the military dynamics of occupation and maintenance of the Roman Empire.

The exceptional preservation of the organic items was possible thanks to the anaerobic conditions at the site, particularly in the waterlogged ditches, with the survival of both the wood and the legibility of the writing for over nearly two millennia [49]. These environmental conditions occur in the presence of specific compounds and microorganisms in the soil, with mechanisms of development that are still not completely understood [49, 50]. However, the archaeological environment led to the formation of vivianite (Fe_3_(PO_4_)_2_ ・8 H2O) on some items, a bluish mineral usually associated with specific environmental contexts [51–53]. It can form in soils with a lack of oxygen, the presence of organic matter, the availability of iron and phosphorus, a low content of sulphur and low pH values [50, 53–56]. When exposed to oxygen during excavation, Fe(II) is converted to Fe(III), giving the characteristic blue colour associated with vivianite [52, 53, 57–59]. In the case of Vindolanda, a decisive aspect for the preservation of organic artefacts was the presence of clay between the different layers of historical activity at the site, which sealed out oxygen [49]. Therefore, the site of Vindolanda is giving new clues about the biomarkers and the microbiological dynamics involved in postdepositional processes and the preservation of archaeological artefacts in soil [49–51].

### 2.2. The Vindolanda Tablets

Among the organic objects in the collection, there are wooden writing tablets, including at least 340 stylus tablets and around 1300 postcard‐sized ink‐written tablets [60]. These numbers are increasing thanks to further discoveries from recent excavations. While the former typology consists of a recyclable wax‐coated wooden tablet inscribed by scratching the wax layer with a metal or bone *stylus* pen, the latter group is known as ‘leaf tablets’ and consists of very thin wooden pieces with writing applied by using ink and a split nib pen. These tablets provide an extraordinary record of daily life, contributing to the reconstruction of the complex network between the Roman auxiliary soldiers and their interactions with the local population [61, 62]. The content ranges from personal letters (health concerns, family matters, social invitations, festival organisations and homework) to military practices (reports, duties, recommendation letters and administrative communications), and economic records (supply lists including food, drinks and clothes). The tablets were discovered in the 1970s during excavations at Vindolanda, when ink traces on thin scraps of wood were first recognised by Robin and Patricia Birley and highlighted by IRR images at the Newcastle University School of Medicine by Alison Rutherford [48]. The collection of these images had been prompted by an urgent need for wood stabilisation and the development of the most suitable cleaning procedures [63], while the IRR images subsequently allowed the tablets to be read by palaeographers. A first attempt at exploring AI systems for text interpretation has been made [64] and, although it has not yet been fully implemented and applied, this will soon be the subject of a doctoral thesis based at the BM and the University of Nottingham. In the last 2 years at the BM, the research project ‘Making History: The making of the Vindolanda wooden writing tablets’ sought to understand their manufacture, the composition of the inks and the best condition for their long‐term preservation [65].

## 3. Material and Methods

### 3.1. Analytical Workflow

A multianalytical approach was planned to outline a suitable systematic protocol for the huge *corpus* of the Vindolanda ink tablets in the BM collection. Dealing with very thin (≤ 1 mm) and fragile objects, found mostly in waterlogged archaeological environments, made it necessary to pursue completely noninvasive methods that might easily be replicated in the future for the periodical evaluation of their preservation. Because of the vulnerability of the tablets, most of the methods were performed without removing them from their Plastazote foam housing to avoid possible mechanical stress and damage during handling. The protocol was tested on a selection of tablets (Table 1). Each tablet is identified by the BM registration number and by a registration number assigned by archaeologists of the VT at the time of their discovery (first and second columns of Table 1, respectively). Images of tablets and their content can be viewed in the BM collection online.

**TABLE 1:** List of ink tablets.

BM reg. n.	VT reg. n.	Document type	Location of the author
1989,0602.66	312	Private document	Vindolanda
1989,0602.69	214	Private document	External
1989,0602.79	185	Military document	Vindolanda
1993,1103.128	649	Private document	Vindolanda
1980,0303.4	190	Military document	Vindolanda
1980,0303.22	250	Private document	Vindolanda
1995,0701.285	575	Military documents	Vindolanda
1980,0303.34	225	Private document	Vindolanda
1995,0701.301	663	Private document	External
1995,0701.319	622	Private document	External
1995,0701.320	588	Military document	Vindolanda
1995,0701.373	628	Private document	Vindolanda
1995,0701.399	643	Private document	External
1986,1001.63	294	Private document	External
1986,1001.223	156	Military document	Vindolanda
1995,0701.182	648	Military document	Vindolanda
1989,0602.71	663	Military document	Vindolanda
1995,0701.401	622	Private document	External
1995,0701.427,429	589	Private document	Vindolanda
1995,0701.216	628	Private document	Vindolanda
1995,0701.9	643	Private document	Vindolanda
1993,1103.12	n/a	n/a	n/a
1993,1103.89	690	Private document	External
1995,0701.114	601	Military document	Vindolanda
1995,0701.159	779	Military document	Vindolanda

The analytical procedure was based on three main steps:

 1^st^ Stage: imaging methods for a preliminary examination of the tablets to choose the most appropriate techniques for the following complementary stages.
 2^nd^ Stage: Raman spectroscopy for the ink characterisation.
 3^rd^ Stage: X‐ray‐based techniques for elemental information on macroscopic and microscopic areas.

For a preliminary visual examination, MBI was used to take colour‐calibrated visible (VIS) images to assess their preservation state, together with IRR images and the elaboration of infrared‐reflected false colour (IRRFC) images for the identification of the ink typologies and the possible presence of mixed inks. As mentioned above, iron‐gall inks and vegetal inks are transparent in the NIR, with a consequent near‐complete disappearance of these in IRR, while carbon‐based inks show no change in opacity [66]. SWIR imaging was then applied to confirm and enhance the carbon black writing [38–40, 67]. Reflectance transformation imaging (RTI) was used to explore the surface texture and to look for possible signs of indentation or scratches that are not visible by standard photography [68, 69].

Subsequently, areas of interest highlighted during the MBI and SWIR investigations were explored at high magnification (up to 200x) by using a digital microscope, which allowed the challenges of focussing on uneven or warped surfaces to be overcome by capturing Z‐stack images, and also enabled 3D models to be created, allowing depth profiling of features of interest. This first imaging step provided sufficient information to facilitate the choice of the best preserved and most representative areas with ink traces and the most appropriate approach for the spectroscopic and elemental methods of analysis.

Raman spectroscopy, as an ideal technique for the identification of carbonaceous matter, was chosen to obtain the molecular fingerprints of the main compounds of the carbon‐based inks to reconstruct the manufacturing process [44, 70–75]. In particular, the deconvolution of the vibrational bands and the application of multivariate analysis allowed the extraction of as much information as possible [74–78]. Complementary information was acquired by recording the elemental characterisation of the inks and any environmental concretions. Specifically, noninvasive investigations were carried out using X‐ray fluorescence (XRF), while scanning electron microscopy with energy‐dispersive X‐ray spectroscopy (SEM‐EDX) was applied on a selection of tablet fragments with ink traces, which were suitable to undergo the variable pressure conditions in the SEM chamber.

**FIGURE 2:**
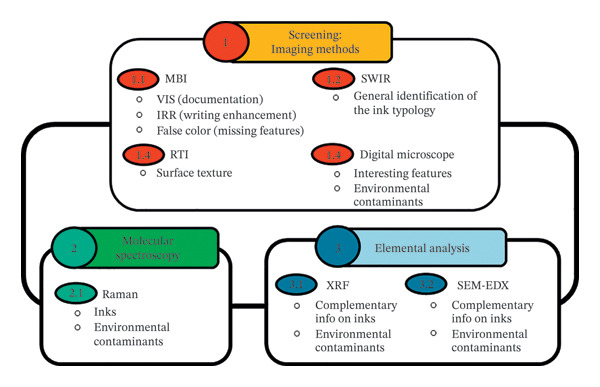
Workflow scheme of the protocol for the ink characterisation.

A summary workflow scheme of the above‐mentioned protocol is summarised in Figure 2.

### 3.2. Imaging Methods

#### 3.2.1. MBI

Two tungsten radiation sources Classic Elinchrom 500 Xenon flashlights were symmetrically positioned at the same height at approximately 45° with respect to the working surface. Before shooting the set of tablets, an image of a uniform reflective whiteboard under the same experimental conditions was taken for the purposes of flat fielding the images.

All images were taken in fully manual mode using a modified Nikon D7000 camera body, with a full sensitivity of the CMOS sensor (c. 300–1000 nm) thanks to the removal of the inbuilt UV‐IR blocking filter. The lens used was a Nikon 35 mm f/1.8II. The VIS and IR‐reflected (IRR) images were taken using, respectively, the X‐Rite CC1 and Schott RG830 bandpass filters in front of the camera lens to select the wavelength range of interest.

The tablets were imaged together with two sets of colour and greyscale references used as internal standards, both adjusted in the same plane of the object’s surface. These references, a Macbeth chart for colour calibration and a reference greyscale with a set of Lambertian black, grey and white tiles, have certified reflectance properties with associated RGB values across the UV‐VIS‐IR spectral range, thus allowing us to obtain pictures with calibrated RGB values in the postprocessing step.

All images were acquired as RAW images and transformed into 3888 × 2592 pixel resolution images in 16‐bit TIFF format using Adobe Photoshop to suppress other automatic algorithms applied from the sensor in the production of RAW files. The postprocessing procedures were carried out using Nip2, an open‐source graphical user interface of the image processing software VIPS. Within Nip2, the plug‐in ‘BM_workspace’ was used for the calibration of the VIS‐reflected and IRR images and the creation of IRRFC images, which were produced by combining VIS and IRR images taken in the same light conditions and focal length [79].

#### 3.2.2. SWIR

An Osiris Imaging System (Opus Instruments, UK) was used to provide 16‐megapixel IRR images with 256 grey levels in the 900–1700 nm range. The camera has an InGaAs line array sensor and a six‐element 150 mm focal length f/5.6–f/45 lens. Depending on the area selection, the image size can vary between 512 × 512 and 4096 × 4096 pixels. Two tungsten Classic Elinchrom 500 Xenon flashlights were used as radiation sources with an approximate position at 45° with respect to the focal axis of the camera.

#### 3.2.3. RTI

Superficial texture details were imaged using an RTI dome system (University of Southampton) consisting of a 1 m diameter dome hemisphere with 76 LED lights (Bridgelux BXRA‐56C1000‐A‐00 LEDs, with a colour temperature of 5600K) and a Nikon 800D camera fixed at its apex. A total of 76 images were recorded, illuminating in turn each LED. The images were processed and combined into the polynomial texture map (ptm) file using the open‐source software RTI Builder (by Cultural Heritage Imaging). The results of the rendering process were displayed using RTI Viewer, where the interactive images can be adjusted by changing the light directions and the filters for texture enhancement.

#### 3.2.4. Digital Microscopy

A Keyence VHX‐5000 digital microscope was used to obtain magnified images (20–200x). The microscope is equipped with a lens VH‐Z 20R, an automated stage VHX‐S 550E and LED‐reflected illumination. 3D observations were realised by moving the *Z*‐axis motorised stage, defining the focus from the lowest point to the highest point of the selected area and taking multiple images at a fixed speed. The series of images at different focal planes is composed in a single 3D image that displays the depth composition, thus giving height information. From these images, it was possible to measure the selected features by tracing a line to extract the profile variation in depth.

### 3.3. Raman Spectroscopy

Raman spectroscopy was carried out with a HORIBA LabRAM HR Evolution Raman spectrometer using green (532 nm) and red (785 nm) lasers for the reference samples and only the green laser on the tablets, with a maximum power of 2.5 and 1 mW at the sample, respectively, a TE air‐cooled electron‐multiplying CCD (EMCCD) detector, operating at −60°C and an Olympus microscope system. The spectra were processed using OriginPro 2018, applying the Peak Analysis function for the band deconvolution, and the Principal Component Analysis (PCA) and the Cluster Analysis tools developed for spectroscopic data within the multivariate analysis section. Further information on the methodology and the analytical conditions employed is provided in the dedicated Section 4.2 *Raman Spectroscopy*.

### 3.4. Elemental Information

#### 3.4.1. XRF

Elemental information was detected by using an Artax XRF spectrometer operating at 50 kV and 500 μA with a collimated beam of 0.65 mm and a counting time of 200 s. Three points of measurement were taken on both the ink areas and the wooden tablet to exclude the elemental contribution from the writing substrate. The analytical lower limit is *Z* ≥ 11.

#### 3.4.2. SEM‐EDX

After the evaluation of the structural suitability of some wooden fragments with ink traces, they were secured to the stage in the vacuum chamber using two pieces of folded silver tape, to avoid any adhesive in direct contact with the fragment. The folded corners of the silver tape helped the identification of the ink lines, which are difficult to recognise due to their isolating properties. Examinations were carried out with a variable pressure VP SEM (Hitachi S‐3700N) using the backscatter electron (BSE) detector at 16 kV and a working distance of 10 mm. The SEM chamber was only partially evacuated, using 40 Pa. The EDX spectra were collected using an Oxford Instruments AZtec EDX spectrometer with a 0 ± 20 keV spectral range, 150 s live time and 2048 channels. AZtec Energy analysis software (Oxford Instruments) was used to process the data.

## 4. Results

### 4.1. Imaging Methods

**FIGURE 3:**
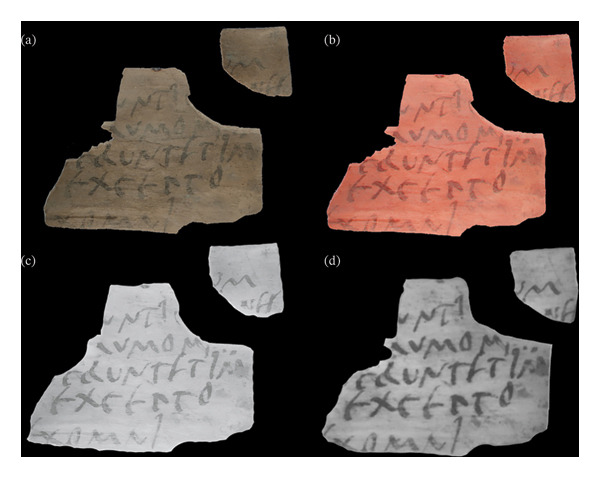
VIS (a), IRRFC (b), IRR (c) and SWIR (d) images of tablet 1995,0701.285 (VT575) indicative of the presence of carbon‐based black ink.

From a preliminary examination, the combination of MBI and SWIR investigations demonstrated that the same typology of ink was used in all the tablets (Figure 3) analysed. In all the IRR images acquired, no changes in opacity and homogeneity were observed in the script, indicative of the presence of carbon‐based inks. Due to the comparative transparency of the wooden substrate to IR wavelengths, the legibility of the writing was significantly improved. The possible use of mixed inks was disregarded after finding that the script in all the corresponding IRRFC images appeared very uniform. The SWIR images further confirmed the use of carbon‐based inks, with improved readability provided by the increase in the contrast between the script and the wooden tablet.

**FIGURE 4:**
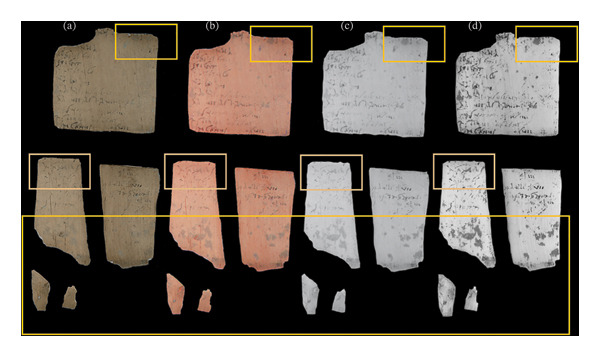
VIS (a), IRRFC (b), IRR (c) and SWIR (d) images of tablet 1989, 0602.71 (VT181). The green areas show the ink was abraded by the presence of encrustations, with dark stains (yellow areas) in the SWIR image due to the presence of environmental remains.

MBI also allowed the detailed documentation of the overall conservation state of the tablets under investigation. The colour‐calibrated VIS images showed the general presence of unevenly distributed areas with a bleached appearance, corresponding to ink loss and writing that appears faded, together with staining and residual encrustations from the depositional context (Figure 4). In the IRRFC images, these areas were highlighted due to their different responses, allowing the easy localisation of degradation effects for further investigation. Moreover, it was also possible to infer dissimilar chemical compositions based on their different optical characteristics. In particular, orange stains and reddish residues were observed to appear yellow or brown in the IRRFC images, whereas bluish powdery encrustations were associated with violet–blue shades. These areas also appeared transparent (light‐coloured) in the IRR and SWIR images, thus lowering their (often disturbing) visual ‘noise’ and contributing to the improved legibility of the writing. Moreover, the readability of the letters affected by external residues was not improved, indicating that the ink was removed by the delamination of the tablet surface induced by the accumulation of encrustations. Indeed, as the IR spectral band can reveal subsurface details with its high penetration in depth with respect to the VIS range, any ink traces below these residues would have been detected. Additionally, in the SWIR range, some areas affected by the presence of environmental remains were highlighted as dark stains, suggesting the possible presence of carbon‐based inclusions (Figure 4).

**FIGURE 5:**
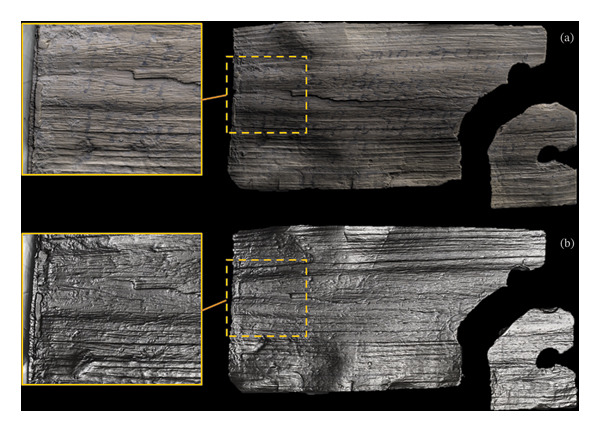
RTI images showing the surface texture (a) and the specular enhancement (b) of tablet 1995,0701.401 (VT642) cut in the radial plane. No traces of indentation from the writing were identified in this tablet. The related close‐up images show the intended cut at the edge.

The final step of the macroscopic phase of the investigation involved the mapping of the surface texture by applying RTI to a small selection of the flatter tablets, whose condition allowed for the safe removal of the top of their foam housing. The polynomial texture mapping (PTM) approach designed for RTI software, based on the calculation of the biquadratic polynomial of each pixel of images taken under varied lighting conditions, allowed the interpolation of an arbitrary light condition to be graphically explored and enhanced by interactively modifying the direction of illumination in the composite images [68]. In the investigated selection, the surface of the tablets appeared to have been affected by delamination effects, with cracks and fractures caused both by the depositional context and the natural readaptation/warping of the wood to the new postexcavation environment. The imaging highlighted that the written lines followed the natural lines created by the radial section of the wood in most of the analysed tablets (Figure 5(a)). In some cases, specular enhancement emphasised topographical features, leading to an improved view of some holes, surface encrustations and deformations that could potentially lead to future instances of cracking (Figure 5(b)). The presence of unintentional scratches was also suggested by their random distribution, while no trace of indentation or elevation was identified in those areas corresponding to writing. Unique intentional cut marks were identified where sharp edges survived on tablet 1995,0701.401 (VT642), as well as traces from the upper wooden layer removed by the environmental context (Figure 5).

The observation of the tablets through imaging methods at progressively smaller scales allowed the identification of the well‐preserved areas of the ink writing, where the 2^nd^ and 3^rd^ stages of the investigation plan were subsequently performed.

### 4.2. Raman Spectroscopy

#### 4.2.1. Guidelines for the Identification of Carbon‐Based Precursors in Raman Spectroscopy

Carbonaceous materials are well known for their two distinctive broadbands around 1300 cm^−1^ (disorder band, known as D1 or D) and 1600 cm^−1^ (graphitic band, indicated as G band) occurring in the first‐order range of the spectrum (1100–1800 cm^−1^); however, evidence for the starting materials and clues about their production can be extracted via further processing. The deconvolution of the bands and the contemporary inspection of some spectral parameters (i.e. position, width and relative intensity) represent the vibrational molecular fingerprint of the raw material [75, 77, 80–82]. The method is sensitive to the detection of changes between crystalline and amorphous structures, represented, respectively, by the graphitic band (G) and the disordered bands, thus allowing information about thermal treatments [72, 83] that have been undergone during production. Further bands, typical of other functional groups in complex compounds, can help with the identification of the raw starting material [84]. The inspection of these parameters, together with the spectral profile and the acquisition of complementary information, can lead to the identification of carbon‐based sources of black materials, which can be classified into at least six main categories [70–74]. Carbon‐based inks can belong to graphite in the class of crystalline carbon‐based phase, or to five macrogroups (microcrystalline graphite in amorphous matrix, coke, charcoal, flame carbons and coal) of amorphous carbon‐based compounds [70–78]. Their main characteristics are described in Table 2.

**TABLE 2:** Typologies of amorphous carbon‐based categories.

Carbon‐based compounds		Origins	Characteristics of the compound
Main category	Main subcategories		
Microcrystalline graphite in an amorphous matrix	Graphite, black earth, shungite and black chalk	Disordered graphite with defective forms of natural origin, including black earths	Thin laminar silvery‐grey flakes with the presence of mineral constituents, i.e. quartz and haematite
Flame carbons	Furnace black, lamp black, peach black, grape black, cherry black, bistre, bistre from wood, atramentum and asphaltum	Originating from soot collected from burning oils, resins or fats, and fruit pits	Clumps of turbostratic particles from tarry materials with a huge variety of additional constituents, depending on the precursor
Cokes	Bone black and ivory black	Solid precursor which fuses upon heating by pyrolysis and graphitisable carbons	Highly variable irregular porous lumps, with the presence of calcium sulphate and phosphate
Charcoals	Wood charcoal and plant charcoal	Solid precursor which maintains its morphology and nongraphitisable carbons	Fragmented flakes with retained structural characteristics, generally containing sodium and potassium salts, carbonates, also iron‐based compounds in the case of vine black
Coals and humic	Van Dyke brown, Cassel brown and bitumen	Organic‐origin rock by natural carbonisation of vegetal remains	Highly variable structures with additional minerals from the soil deposit

Following the procedure described by Coccato et al. and Tomasini et al. [71, 74, 78], the data references available in the scientific literature were enlarged by analysing the carbon‐based materials available in the wide reference collection of standard pigments of the BM. This database includes known unbranded materials, pigments supplied by Cornelissen and Son, and by Kremer. In this case, only the plant‐based and vine black references were bought by Cornelissen and Son and Kremer, respectively (Table 3).

**TABLE 3:** Parameters of carbon‐based pigments in the BM reference collection.

Reference	Category	D1 band (cm^−1^)	G band (cm^−1^)	Description	Other bands (cm^−1^)
WOOD	Charcoal	**1319 ± 2**	**1578 ± 3**	Glassy translucent particles, flake‐like elongated material and sharp edges	**Around 1220 (D4), possible broadband in the range 776-885**
		*1358* ** ±** * 5*	*1589* ** ±** * 2*		1227–1238 (D4), 1480–1541(D3)
PLANT		**1328 ± 5**	**1589 ± 1**	Glassy translucent particles, flake‐like elongated material and sharp edges	**1217 (D4)**
		*1344* ** ±** * 9*	*1592 ± 2*		1222–1334 (D4), 1520–1569 (D3), 417 (traces of spinel MgAl_2_O_4_), possible carbonyl bond in the range 1725–1727

VINE	Flame carbon	**1318 ± 1**	**1584 ± 1**	Tarry material with reddish and brownish particles, unstable under low‐laser power	**1220 (D4), 1499 (D3)**
		*1362* ** ±** * 7*	*1590* ** ±** * 4*		1358 (D4), 1580 (D3), 210–272 (traces of haematite), 682 (traces of iron‐based compound)
LAMP_1		**1305 ± 1**	**1589 ± 1**	Crumb‐like very opaque material, with rounded grains and turbostratic structure	**Noisy**
		*1336* ** ±** * 1*	*1588* ** ±** * 1*		1337 (D4), 1555 (D3), broadband in the range 730–790
LAMP_2		**1306 ± 3**	**1588 ± 3**	Crumb‐like very opaque material with rounded grains and turbostratic structure	**Noisy**
		*1338*±	*1588*±		1338 (D4), 1558 (D3), very broadband around 760–780

IVORY_1	**Coke**	**1317 ± 3**	**1593 ± 1**	Dusty opaque fine particles, greyish and colourless particles, jagged edges	**1510 (D4)**
		*1342* ** ±** * 2*	*1593* ** ±** * 5*		1223–1260 (D4), 1521–1531 (D3), broad peaks at 764 and 954, possible peak at 1078
IVORY_2		**1317 ± 5**	**1588 ± 3**	Dusty opaque fine particles, greyish and colourless particles, jagged edges	**1545 (D3), possible broadband in the range 760-786**
		*1350* ** ±** * 8*	*1598* ** ±** * 7*		1214–1337 (D4), 1485–1535 (D3), peaks in the ranges 951–961, and 1073–1084

*Note:* Bold values were acquired using the 785 nm laser, and values in italics were collected using the 532 nm laser.

**FIGURE 6:**
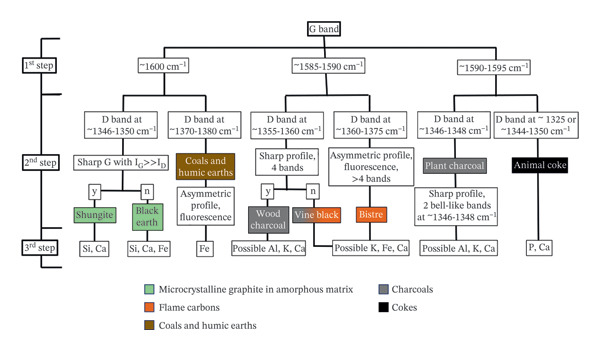
Workflow for the identification of the various sources of carbon black from Raman spectra.

A procedure for carbon black identification from Raman spectra, using a specific decision tree, was adopted, as summarised in Figure 6. It should be noted that the following instructions have been verified using a green laser at 532 nm. The ID/IG ratio is proportional to (ID/IG) × E_0_ (eV)^4^, with E_0_ referring to the laser energy, because the process involves parameters evolving as the fourth power of the laser wavelength [80, 84]. Consequently, the same carbon‐based compound can yield different spectral profiles regarding the number of bands, their intensities and the wavenumber values of their maxima, depending on the laser used, thus making it difficult to compare data obtained in different conditions.

When starting with the identification, the G band represents the first feature for observation (step 1 of Figure 6), followed by the position of the D band and the spectrum profile (step 2 of Figure 6).

 Step 1a: If the G band is positioned at ∼1600 cm^−1^
 This relates to the in‐plane vibrations of sp^2^‐bonded carbons, with a sharp peak at ∼1600 cm^−1^ in graphite and in ordered materials, and it is also the case for black earth and black chalk, which are characterised by microcrystalline carbons, and disordered carbons, including coals and humic earth.
 Step 2a: Check the D band and the spectral profile
 The use of well‐ordered carbons with a graphitic sheet structure dispersed in an amorphous matrix (such as that of naturally occurring shungite) can be confirmed by the presence of a low‐intensity D1 disordered band at ∼1346–1350 cm^−1^. The exact position will depend on the mixture of sp^3^‐, sp^2^‐ and sp^1^‐hybridised carbon bonds present, which is related to changes in the ring/chain structures and their breakdown within the increase of in‐plane defects and heteroatoms [74, 80]. The D2 band, usually occurring at ∼1620 cm^−1^ in highly ordered structures, is often not distinguishable from the G band in these types of carbon‐based structures [41]. However, in graphitic carbons, the G band broadens and shifts down to ∼1580 cm^−1^ under high thermal conditions with a consequent increase in the height and width of the D1, D2 and D3 bands, with the D1 band maximum generally positioned at ∼1350 cm^−1^. If the G band remains at ∼1600 cm^−1^ and the D1 band is sharp and high in intensity, this is characteristic of black earths. Additionally, other associated bands belonging to quartz, calcite and haematite can usually be detected in this case.
 In coals, the G and D1 band positions shift to high wavenumbers, so even if the G band appears at ∼1600 cm^−1^, it is generally easy to recognise them due to the very asymmetric D1 band around ∼1380 cm^−1^ and usually intense fluorescence background [74, 85]. Being sedimentary rocks with organic origins, coals are usually composed of humic earth together with quartz, oxides and carbonates, which can also be identified in Raman spectroscopy and confirmed with complementary analyses.
 Step 1b: If the G band is positioned at ∼1585‐1590 cm^−1^
 This is characteristic of charcoals, which have sharp bands, but depending on the content of lignin, cellulose, hemicellulose and the different carbon‐to‐oxygen ratio microstructure, the final spec can change. It should be noted that it is not possible to make a species identification from the botanical point of view, because Raman spectroscopy analyses a matrix of amorphous carbon with cross‐linking bonds, which can be common to several species. However, based on available references, the term ‘wood’ has been assigned to charred fragments of woody trunk, and the term ‘plant’ to a mixture of charred grass and straw, where the cellulose content remains more visible in Raman spectroscopy. Wood charcoal can be surmised by the presence of the G band at ∼1585‐1590 cm‐1, as well as bistre, a subgroup of flame carbons that includes carbonaceous matter from soot, tarry materials, vegetal exudates and fruit stones. For plant‐based charcoal, step 1c.
 Step 2b: Check the D1 band, the possible presence of further disorder bands (> 4) and their symmetry
 Raman spectra of wood charcoals are sharp and recognisable by their D1 bands positioned at ∼1355 cm^−1^. The deconvolution of D3 and D4 is always possible, but their position depends on possible methylene groups, defects of oxygen‐rich precursors and the different levels of amorphisation and hybridisation occurring during carbonisation. Other bands may be present between 1000 and 1320 cm^−1^, but only in case of a high degree of disorder. As wood charcoal consists mainly of carbonised lignin, the amorphous mixture contains dispersed graphitic sheet microstructures and polyaromatic stacks with a consequent resistance to thermal degradation [77, 78, 84, 86]. Therefore, as a result of the preservation of the solid‐phase structure, the profile is more symmetric than bistre.
 The category of flame carbons (often generally termed ‘soot’) has high variability, depending on the raw material. Apart from a few cases (such as furnace black), flame carbons are generally characterised by poorly ordered structures with a D1 band at ∼1360‐1375 cm^−1^, several asymmetric disordered bands, an increase of fluorescence, the growth of the D4 band and a broad D3 band as the result of the structure fragmentation [72, 74]. This is the case with bistre, whose G band at ∼1590 cm^−1^ can be less intense than the D3 band and can be found together with an intense and highly broadened D1 at ∼1360 cm^−1^ in soot. In this group, the D3 and D4 bands can also be related to defects outside of the carbon plane and to the particular nature of the source materials. Indeed, soot can be the result of fruit pits or tarry residues of burnt wood with a possible further boiling step [74]. In this last case, the term ‘wood from bistre’ has been used in the following sections. Vine black, a well‐known black pigment widely used since antiquity and based on a mix of charred vine twigs, lees and grapes, is a type of bistre. Its D1 band is usually positioned at ∼1360 cm^−1^, and a total of four disordered bands can be detected.
 Step 1c: If the G band is positioned at ∼1590–1595 cm^−1^
 It is indicative of vegetal materials with a high cellulose content, such as in plant charcoal, where the G band shifts up to ∼1590‐1595 cm^−1^. Nevertheless, this is also the position of the G band in case of bone black.
 Step 2c: Check the presence of a bell‐like profile
 In plant charcoal, the D1 band moves down to ∼1346‐1348 cm^−1^. During the deconvolution, the D4 band is usually close to or aligned with the maximum of the D1 band, inducing a bell‐like shape in the convoluted D band. Two more bands can be fitted between 1070 cm^−1^ and 1280 cm^−1^, increasing and changing with the transition from microcrystalline cellulose through to modifications in crystal structure until finally becoming amorphous cellulose III [86, 87]. The disordered bands can easily broaden because the degree of amorphisation increases with cellulose content. With increasing firing temperature, cellulose undergoes water desorption, dehydration and thermal scission of the glycosidic linkages, ethers and C‐C bonds [70, 72, 83–85, 87–89]. During carbonisation, cellulose is converted into disordered graphene‐based nanoporous structures in an amorphous phase, so that the profile remains sharp.
 The case of the plant‐based charcoals is an important example of how the deconvolution of the disordered bands can help to distinguish between degraded charcoals and other carbon‐based categories. For instance, plant‐based charcoal and furnace black, a particular subgroup of flame carbons, can have similar D1 and G maxima [74]. However, coming from the incomplete combustion of hydrocarbons from oil or bituminous materials, different contributions mainly from alkyl groups with additional broad peaks can be identified in the deconvolution of furnace black spectra [74].
 Conversely, the identification of animal cokes can be less straightforward. The G band is usually reported at ∼1595 cm^−1^, while the D band maxima can be at ∼1325 or between ∼1344 and ∼1350 cm^−1^, thus producing profiles similar to the previously described categories. In cokes, the D bands are related to the changes between the mineral constituents and the collagen contribution, as represented by bands due to amide I, amide III and C‐H bending [90]. The D1 band can easily shift because of the graphitisable nature of this typology, where the precursor decomposes in a liquid/plastic texture immediately before carbonisation [74]. This is also influenced by the sample granulometry, because the material reduced to powder allows a high disorder structure to be achieved much more easily, with a corresponding shift towards higher wavenumbers (Figure 6) [74]. Signals from phosphates can be detected, with a major peak at ∼957 cm^−1^, which is unfortunately not always clearly identifiable in bone black [74]. Complementary information must be acquired for verification.
 Step 3: Complementary information
 Apart from the criteria related to the spectral profile described in the first and second steps (parameters involving the D1 and G bands, number of disorder bands and their positions in the consideration of the shift due to the possible chemical source), a third step to support the source identification involves the use of additional techniques to look for the presence of specific elements or minerals (Figure 6).

Finally, multivariate analysis can be performed to extract further information and double‐check the identification. These stages are described in the following section.

#### 4.2.2. Identification of the Carbon‐Based Ink in the Vindolanda Tablets

**FIGURE 7:**
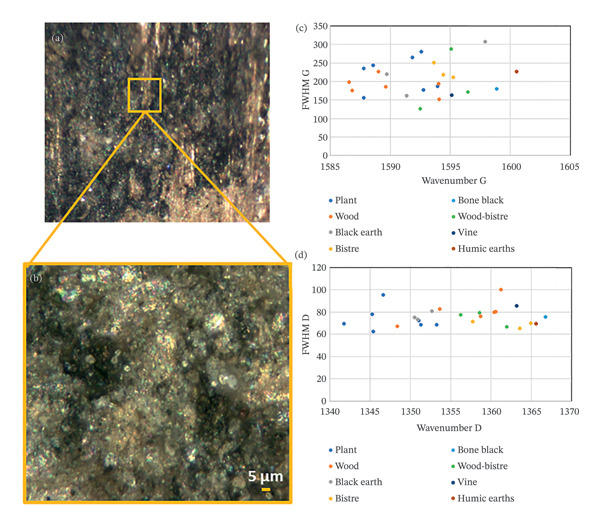
Micro‐Raman spectroscopy applied to the ink particles (a, b) and identification of the carbon‐based sources using the G (c) and D1 (d) wavenumbers at their maximum intensities.

Having surveyed the carbon‐based typology with imaging methods and identified the best preserved areas under the digital microscope, micro‐Raman spectroscopy was used for the characterisation of the source of carbon‐based black materials. Increasing the magnification up to 150X, the ink is distributed as rounded agglomerates with traces of binder in uneven stripes depending on the tablet surface (Figure 7(a)). The presence of micrometric fragments from burnt vegetal remains from the archaeological environment was not problematic, as they can be distinguished by their jagged flakes, unlike the rounded and compact particles of the ink‐containing areas.

The extraction of the characteristic parameters (maxima wavenumbers, full width at half maximum and relative intensity of D1 and G bands) was performed by applying conventional fitting procedures with the OriginPro 2018 software package.

The protocol is described by the following steps:

• Normalisation to the 0‐1 range, performed to have comparable data with independence from the scale difference.
• Cropping the spectra in the 1000–1750 cm^−1^ range, to avoid misconceptions from environmental contaminants and other compounds.
• Creation of a baseline, using an interpolation line.
• Addition of the deconvolution bands.
• Looking for hidden peaks in the second derivative residuals until reaching a random pattern of the residual data [91].

Regarding the typology of deconvoluted bands, the D1 and G bands are commonly fitted using Gaussian and Lorentzian functions, respectively [73]. However, for the dataset analysed, the Gaussian function was better suited for fitting the G bands, as this choice led to lower residuals [92, 93]. In heterogeneous environments, broadening mechanisms can convert an intrinsic Lorentzian line shape, used to model pure vibrational modes, into a Gaussian one [94]. The Voigt function, representing a convolution of a Gaussian and a Lorentzian function, was discarded as being less effective in the reduction of the chi‐squared parameter and the final residual sum [92, 93].

Table S1 (Supporting Information) shows the mean values of the extracted parameters together with their corresponding statistical deviation. The identification of the sources was performed considering the deconvolution, the number of extracted bands, the spectral profile and the maxima wavenumbers of the D1 and G bands.

The characteristics of the extracted D1 and G bands are illustrated in Figure 7, where the FWHM of the G and D bands is plotted versus their relative positions. The comparison of these features helped the recognition of the carbon‐based sources of black materials, as shown in the previous section. The identification of the carbon black typology based on the results of Raman spectroscopy is listed in Table 4, together with complementary information obtained by applying XRF and SEM‐EDX on a selection of tablets. The XRF and SEM‐EDX outcomes are discussed separately in Section 4.2.3.

**TABLE 4:** Resume of the results obtained applying Raman spectroscopy, X‐ray fluorescence (XRF) and scanning electron microscopy with energy‐dispersive X‐ray spectroscopy (SEM‐EDX).

BM reg. n.	VT reg. n.	Raman	XRF	SEM‐EDX
1989,0602.66	312	Plant charcoals	—	—
1989,0602.69	214	Mixed charcoals, preponderantly wood‐based	Si^tr^, K	—
1989,0602.79	185	Bistre	S, Fe^tr^	—
1993,1103.128	649	Bistre	—	—
1980,0303.4	190	Plant charcoals	S^tr^, Ca	—
1980,0303.22	250	Wood charcoals	—	—
1995,0701.285	575	Mixed charcoals, preponderantly plant‐based	Ca	—
1980,0303.34	225	Mixed charcoals, preponderantly wood‐based	K, Ca, Fe^tr^	—
1995,0701.301	663	Bistre	K, Fe	—
1995,0701.319	622	Vine black	Fe	—
1995,0701.320	588	Bistre from wood	—	Al, Si, K^tr^, Ca^tr^, Fe^tr^
1995,0701.373	628	Mixed compound based on flame carbons, preponderantly bistre from wood, and wood	Ca, Mn^tr^, Fe	—
1995,0701.399	643	Wood chars	Fe^tr^	Al, Si, K^tr^, Ca^tr^, Fe^tr^
1986,1001.63	294	Wood chars	—	—
1986,1001.223	156	Plant chars	—	—
1995,0701.182	648	Bone black	P, Ca	—
1989,0602.71	663	Black earth	—	—
1995,0701.401	622	Plant chars	Si^tr^, K, Fe^tr^	—
1995,0701.427, 429	589	Flame carbons (possibly bistre from wood)	Ca, Mn^tr^, Fe	—
		Humic earth	Fe	—
1995,0701.216	628	Bistre from wood	—	—
1995,0701.9	670	Bistre from wood	Si^tr^, S^tr^, K	Al, Si, K
1993,1103.12	n/a	Plant chars	—	—
1993,1103.89	690	Black earth	—	—
1995,0701.114	601	Bistre from wood	—	—
1995,0701.159	779	Bistre from wood	—	—

*Note:* tr = trace element.

**FIGURE 8:**
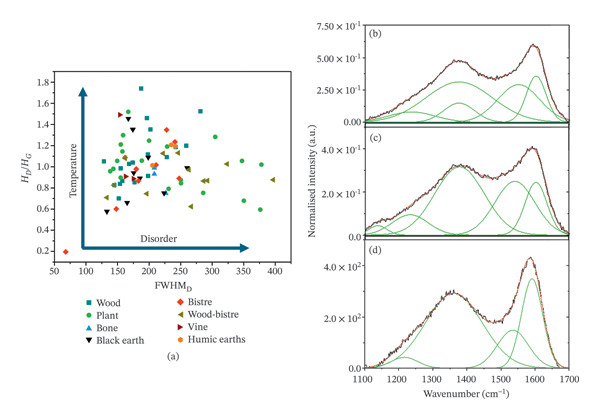
Extraction of parameters from the Raman spectra: qualitative thermal indications (a) given by the intensity ratio of the D1 and G bands with respect to the relative FWHM_D_, and band deconvolution of plant‐based charcoal (b), vine black (c) and wood charcoal (d) of the Raman vibrational modes of three different carbon‐based materials.

Five main groups were identified: charcoals, bistre, humic earth, black earth and bone black. In the char category, plant‐based charcoals showed the lowest frequencies of the D band and high frequencies of the G band. Regarding flame carbons, bistre was detected as a result of further bands related to hydrocarbons. However, some compounds identified as bistre can presumably come from wood, due to a typical profile of bistre with a broadened D band but a lack of further bands in the 1000–1300 cm^−1^ range. Among the bistre typology, a single case of vine black was identified. In other categories, humic earth, bone black and black earth are also isolated cases. Regarding tablets 1995,0701.427, 429 (VT588), the mixture of flame carbons, possibly bistre, and humic earth was identified in the two subgroups of fragments (427 and 429, respectively), in this case, on different examples of handwriting. Examples of the deconvolution of the spectral profile for the identification of the carbon black sources are given in Figure 8.

Further information can be found from the extracted parameters, as the disorder depends on thermal degradation. Indeed, the mechanism of amorphisation of the carbon‐based compounds depends on the starting composition [12, 13]. Considering that systematic studies in the literature on the molecular changes with temperature have mostly only considered graphite and graphene [81, 82], it is not possible to extrapolate the exact temperature for the firing conditions. The intensity ratio (*I*_*D*_/*I*_*G*_) of the D and G bands and the FWHM_D_ can give us clues about the heating conditions [82, 83]. According to Figure 8(a), the general intensity ratio (*I*_*D*_/*I*_*G*_) is close to 1, implying the different starting compounds were burnt to high temperatures, inducing high disorder. The variability of the data can be related to a lack of control of the firing conditions and the different starting compounds. The broadening of the D band and the consequent enlargement of its FWHM_D_ at lower *I*_*D*_/*I*_*G*_ are associated with plant‐based charcoals. The high variability of the FWHM_D_ for plant‐based charcoal depends on the cellulose content and its degree of organisation, which can depend on the different anatomical vegetal structures, being differently impacted by the heating conditions. Lignin aromaticity determines the thermal resistance in wood‐based charcoal and the retention of the overall structure [77, 86, 95]. This is reflected in a sharper D band for wood charcoals with respect to plant‐based charcoals (Figure 8(a)). The implication of this information on the firing conditions will be discussed in more detail in relation to the archaeological context in Section 4.2.4.

As part of the development of a proper protocol, the application of multivariate analysis with unsupervised learning methods was tested to verify their support for the identification of different carbon‐based inks in the case of archaeological items with a noninvasive procedure.

Before applying PCA, the statistical significance of the data for a possible differentiation was verified, considering the similarity in the spectra. The two‐way ANOVA parametric test was applied to assess whether the data variance was sufficient. A slight statistical difference was achieved with a *p* value of zero, meaning the data are similar, as expected, but the variance for testing PCA was adequate. Indeed, with a value of *p* < 0.05, it is still possible to reject the null hypothesis, which states the populations have the same mean.

PCA was carried out using the PCA application designed in OriginPro 2018 for chemometric spectra. As presumed from the low statistical difference, the PCA understanding is not completely straightforward due to the presence of subgroups with similar positions of the D1 and G bands. The orientation of the data can be explained considering the categories of carbon black materials, discussed in the previous Section 4.2.1. It is possible to visualise a directional trend for plant‐based and wood‐based charcoals following a different orientation with respect to the bistre category, which includes vine black and bistre from wood (Figure 9). Bone black can be identified as a different group between charcoals and bistre, possibly due to the bituminous nature of the black coal coming from burnt animal coke. Some points from ink identified as wood charcoal fall in the bistre area (Figure 9), due to possible mixtures with bistre from wood and highly disordered carbon black based on wood with increased asymmetry and blue shift, both comparable with bistre. Despite including the bistre typology, some nonidentified flame carbons fall in the same area as the charcoals, probably due to tarry materials with band wavenumbers close to plant charcoals, such as a hypothetical furnace black [74], together with humic earths and black earths. Regarding this last class, further points would be needed for an improved statistical picture because of the huge variety of this category related to different minerals naturally occurring in the compound, including exudes from salt pits [2].

FIGURE 9Application of multivariate analysis using principal component analysis (PCA) for spectral data (a) and hierarchical cluster analysis (b).

**Figure:**
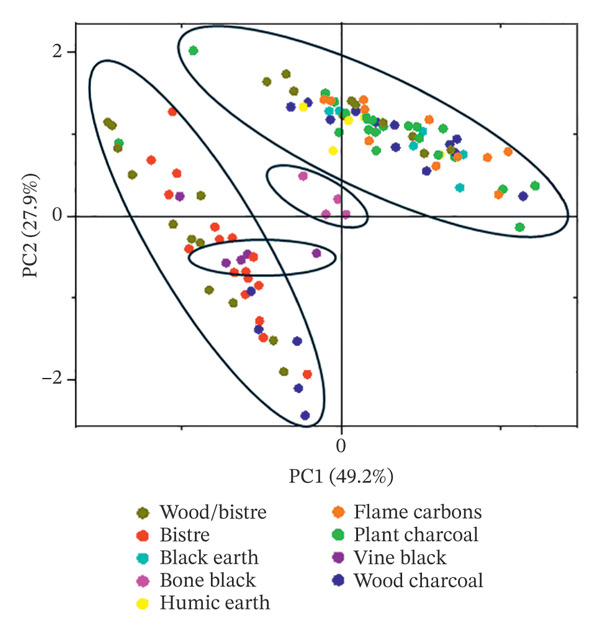
(a)

**Figure:**
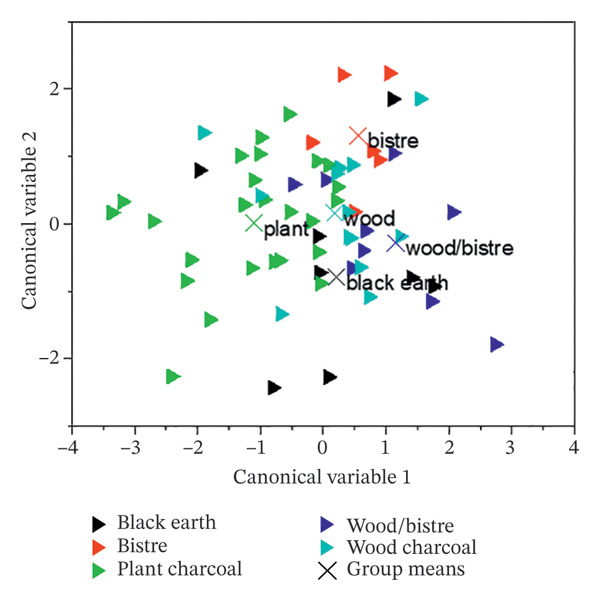
(b)

Although PCA might not be so clear due to the vegetal source as the preponderant raw materials, it can still be helpful to differentiate among charcoals and bistre and to explore the data distribution of the analysed set. As mixtures of different carbon‐based sources can be found in the same ink, the identification of the raw material can be difficult if performed on the average values of the wavenumbers at the maxima intensities. PCA is recommended as a tool for data revision. A mixture of wood charcoal and bistre from wood can be presumed because of the displacement between some points, such as tablets 1989,0602.69 (VT214), 1995,0701.373 (VT628) and 1980,0303.34 (VT225). The presence of mixed carbon blacks could have presumably occurred by changing different batches of different burnt compounds or by making a specific choice.

Hierarchical (or canonical) cluster analysis (CCA) was another multivariate method used to test the presence of groups based on similarities using OriginPro 2018 (Figure 9(b)) and to double‐check some sources identified. In this case, the further neighbour was applied as the cluster method, together with the Euclidean squared distance. This tool worked better with charcoals. Plant‐based and wood‐based charcoals are close to each other, but with two different trends. The bistre, presumably related to wood, overlaps the wood charcoals, while black earth is spread due to its high variability.

The identification of carbon‐based materials has to take into account all parameters of band deconvolution extraction (D1 and G band positions, bandwidth and number of bands), while other methods, such as the explored ones (PCA and CCA), should be used to support and review the categories. PCA can be a tool for differentiating bistre and charcoal typologies, while CCA can be useful in exploring and helping the identification of plant charcoals and wood charcoals.

#### 4.2.3. Information From Elemental Composition Analysis

The investigation of elemental composition using XRF (Table 4, Section 4.2.2) allowed us to double‐check the identification on some ink tablets. The difference in XRF and SEM‐EDX results listed in Table 4 is also due to the conditions of analysis. Light elements, such as aluminium and silicon, were not detected by Artax because the analyses were carried out under normal environmental conditions, without the use of a helium flux, which was precluded by the fragility of the tablets’ surfaces. On the contrary, analysis of tablet fragments containing traces of ink inside the low‐pressure vacuum chamber allowed the detection of low‐Z elements.

For each spectrum, a comparison with the elements and the relative intensities of their peaks was performed with the results obtained on the wood substrate, in areas without ink traces or any major encrustations. This step was necessary not only to identify the possible contribution from wood, but also from other possible contaminants, such as encrustation traces that were not visible at macroscopic levels. In this regard, SEM‐EDX analysis would be more efficient, as it is possible to select micro‐areas of ink, minimising the contribution of wood, and also avoiding areas with micrometric contaminants from the excavation environment. This method could only be safely applied to a small number of tablet fragments that were sufficiently stable from the conservation point of view to withstand variable pressure conditions.

An example of the complementary information given by the application of Raman spectroscopy, XRF and SEM‐EDX is given in Figure 10. Despite the similarity of the Raman spectra for vine black (1995,0701.319 (VT622)) and bistre from wood (1995,0701.9 (VT670)), a clear difference is noted in the XRF results (Figure 10(a.1) and (b.1)), with the presence of iron for the former and potassium for the latter. The SEM micrograph (Figure 10(b.3)) shows the same striped pattern of the ink identified in the confocal microscope (Figure 7(a)), with the presence of small particles of encrustations. The EDX spectrum obtained from the micrometric area of the ink highlighted the additional presence of aluminium and silicon, confirming the use of bistre from wood (Figure 10(b.4)).

FIGURE 10Complementary information obtained on (1995,0701.319 (VT622)) (a) and (1995,0709 (VT670)) (b) consisting of vine black and bistre from wood, respectively. Despite the similarity of the Raman spectra profile (vine black, a.2; bistre from wood, b.2), the XRF spectra confirmed the considerable presence of iron (a.1) for the vine black and the presence of potassium (b.1) for the bistre from wood. The EDX spectrum (b.4) obtained from the SEM micrograph (b.3) allowed the detection of further light elements (aluminium and silicon) in addition to potassium. Other visible elements correspond to the substrate.

**Figure:**
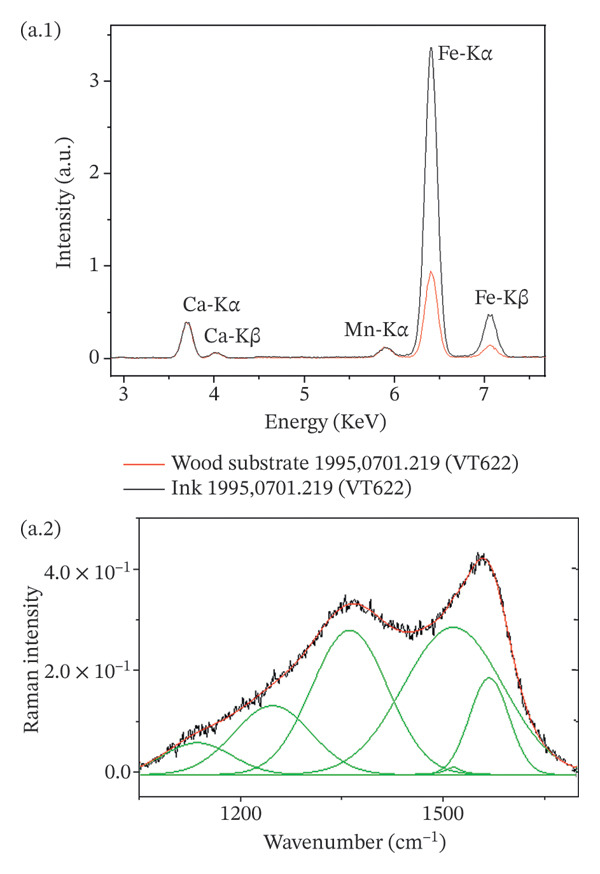
(a)

**Figure:**
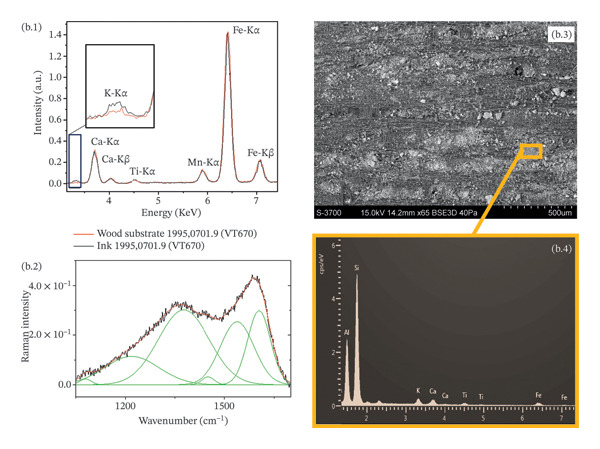
(b)

Bone black on 1995,0701.182 (VT648) was confirmed due to the presence of phosphorus and calcium. Potassium and calcium were present in the vegetal‐based charcoals, while iron was found in the ink based on humic earth. The detection of iron, potassium and sulphur supported the use of bistre. On a micrometric level, due to the high sensitivity of SEM‐EDX for light elements, aluminium and silicon were also detected in tablets 1995,0701.320 (VT588), 1995,0701.399 (VT643), based on wood charcoals and bistre from wood, respectively. Traces of calcium and iron could also possibly come from the environment.

With regard to the encrustations identified during the imaging step of the investigation, the presence of haematite, calcium, quartz and albite found in the spectroscopic phase of analysis was confirmed by the complementary detection of high amounts of iron, quartz and calcium in reddish and greyish areas. The black particles with vivianite and meta‐vivianite were based on manganese, with traces of titanium and potassium, especially in the light‐bluish and greyish areas [96]. It was not possible to assign the presence of sulphur to a specific compound or condition, likely due to the localised effects of environmental conditions and the dynamics of vivianite formation.

#### 4.2.4. Discussion

The obtained parameters on the analysis of inks from a selection of tablets from Vindolanda have allowed the identification of charcoals as the most abundant source. Within this typology, two subgroups consisting of plant‐based and wood‐based charcoals were found, in addition to bistre and possibly bistre from wood. These findings are in agreement with the materials described by Pliny the Elder and Vitruvius for carbon black inks [2, 18, 19], and, indeed, both authors attested soot produced by burning resins and charcoals from wood as the main sources of black inks.

Only tablet 1995,0701.319 (VT622), the content of which tells us that it was written outside Vindolanda (probably at Briga, a fort somewhere nearby) [97], was identified as possible vine black due to its asymmetric spectral profile and the deconvolution of numerous disordered bands (Figure 8(c)). Indeed, the writer was *Aelius Brocchus*, a *praefectus*, a commanding role held by Roman citizens. Regarding its production, vine black was reported as a possible Roman black ink, obtained by cooking in an oven and grinding the charred materials obtained from wine lees in a mortar, to obtain a bluish black ink [2, 19]. In this respect, it is necessary to clarify that no references of other carbon black obtained from other types of vines that were not grapevines have been found. Therefore, it is not certain if ivy vine, native to Britain, may have a similar Raman profile. Nevertheless, it is intriguing that this is the sole case that has been discovered within the analysed set.

A few tablets were classified as having used inks based on black earth, while only one (tablet 1995,0701.182 (VT648)) was found to be related to bone black, despite an unclear detection of the band around 965 cm^−1^, characteristic of the phosphate‐stretching mode of molecular vibration, but confirmed using XRF. Even in this case, such materials were described in the *Naturalis Historia* as possible compounds for black pigments but reported as problematic and limited in use [2, 19]. In the 35^th^ book, Pliny the Elder also mentioned the use of earth and exudates, such as the brine in salt pits, while bone black is said to be troublesome due to the occurrence of the desecration of graves to obtain charred materials [2, 19]. Tablet 1995,0701.427, 429 (VT589) was revealed to contain a mixture of flame carbons, possibly from bistre, and humic earth. The two ink typologies seem to be related to two separate writing events in different scripts. A draft was realised behind an account list, thus reusing the tablet.

A possible mixture with the overlapped groups of materials cannot be excluded when taking into account the testimonies of Vitruvius and Pliny the Elder. They referred to the practice of intentional adulteration of black paints obtained from wood and pine pitch with a mixture of remaining soots. In the 7^th^ book of the *De Architectura,* Vitruvius spoke about ink production using the word ‘workshop’ (*officina*), where the raw material was burnt with an intense fire and then collected from the walls and the vault of the furnace [2, 19]. During this practice, quartz, carbonates and inorganic materials from the furnace could have also been included when the material was harvested from the furnace walls. Regarding the furnace, the author specified it was completely sealed while burning the raw materials and that the area of production was built like a ‘Spartan sweating room’ (*laconicum*). In the 5^th^ book of the *Materia medica*, Dioscorides confirmed the use of mixed inks [18]. He attested that the extraction of carbon black to be used as ink was carried out in factories where glassmakers and goldsmiths worked [2, 18]. This information is in agreement with the *I*_*D*_/*I*_*G*_ ratio value (∼1) and the possible involvement of high furnace temperature, which led to such a degree of disorder in the compounds. Considering Dioscorides suggested the ink manufacture in relation to glassmaking and goldsmithing, the production of carbon‐based compounds used for inks in a Roman fort might be related to blacksmithing, possibly with the view of recycling the available materials. Roman legions had *immunes*, a group of soldiers that included a wide range of people having craft and artisan skills to contribute to self‐sufficiency in the garrison. Iron and lead were reported in the content of the Vindolanda tablets (i.e. 1989,0602.75 (VT182)) in the list of supplies of the Vindolanda fort, and evidence of metalworking, such as slags, broken crucibles, ashes and charcoals, was found in the archaeological site. Although the contents of the tablets provide detailed information on daily life in the fort, outlining supply routes and inventories of materials and objects, it must be noted that no mentions of inks are present, thus supporting the idea of local production. The rare presence of lamps found in the Vindolanda fort is reflected in the absence of ink‐based lampblack.

**FIGURE 11:**
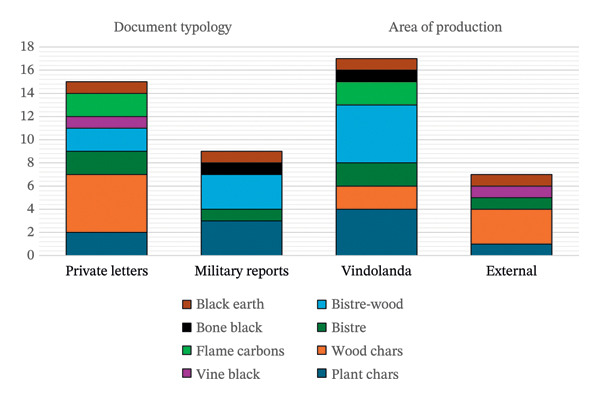
Distribution of the different carbon‐based compounds depending on the document typology, area of production and period of the fort.

Considering a possible differentiation in the hue and opacity of the ink according to the raw material, the possible intentional use of a specific compound rather than another was investigated. To illustrate this, the classification was given using the predominant compound in the case of mixed compounds. In literature describing practices in the ancient world, wood‐based charcoal and bistre were considered to have the best ink quality [2, 18, 19]. These compounds were predominant for private use, possibly because of the disposable role of military documents which often included tasks that had been accomplished or changed (such as the *renuntiae,* a technical term present in some tablets belonging to the military report category, used to mark completed tasks and finished assignments [60]) (Figure 11). Wood charcoal was the major category found in tablets coming from external forts in the form of private letters (Figure 11). Unfortunately, the number of examined tablets does not cover sufficiently high statistics for verifying the impact of certain compounds on the different periods of construction of the archaeological site.

The results of this study reflect exactly the materials described in ancient literature [18, 19]. It is also noteworthy that while in Herculaneum and Egypt, there is already a change in the composition [3, 28], including metal‐based compounds added to carbon black, at the borders of the Roman Empire, the legions proceeded with the traditional practice of production.

## 5. Conclusion

The extraordinary number of well‐preserved tablets found in Vindolanda has given a remarkable opportunity to investigate the ink production at the edge of the Roman Empire, when different practices were arising in the Mediterranean area. A multianalytical approach was tested on a selection of Vindolanda tablets to characterise black ink writing and to find further clues about the ink production and life in a Roman fort.

As the imaging methods (MBI, SWIR and digital microscopy) highlighted the presence of carbon black, a Raman spectroscopy protocol for the identification of carbon‐based precursors was explored, enlarged and applied to extract further information, with complementary elemental characterisation (XRF and SEM‐EDX). The procedure adopted has highlighted the necessary steps for the identification of different carbon black typologies, together with the supplementary use of multivariate methods to double‐check the same carbon‐based categories. The results, which are in agreement with the sources mentioned in early accounts, showed the potential of this method for the analysis of carbon‐based inks and the need to test additional reference materials to advance the subgroups of charcoal and flame carbons.

The extension of this multianalytical procedure on a larger set of tablets from Vindolanda has the potential to elucidate, in future work, if there was a homogeneous practice for ink production or a geographic dependence, as well as tracing the timescale of the development of ink manufacturing and possible preferences of the carbon‐based compounds (such as plant and wood charcoal, bistre, and bistre from wood) within the different building phases of the fort [98, 99].

## Author Contributions

G.V.: data curation; formal analysis; investigation; methodology; validation; visualisation; writing–original draft; and writing–review and editing. J.D.: conceptualisation; investigation; methodology; validation; supervision; visualisation; writing–original draft; and writing–review and editing. R.H.: conceptualisation; validation; project administration; supervision; and writing–review and editing. C.R.C.: conceptualisation; validation; project administration; supervision; and writing–review and editing.

## Funding

The study was funded by Augmentum.

## Disclosure

All authors have read and agreed to the published version of the manuscript.

## Conflicts of Interest

The authors declare no conflicts of interest.

## Supporting Information

Table S1: Deconvoluted Raman parameters with the related standard deviations (Supporting–G and D bands).

## Supporting information


**Supporting Information** Additional supporting information can be found online in the Supporting Information section.

## Data Availability

The data that support the findings of this study are available from the corresponding author upon reasonable request.
