# Supplementary material for: The Making of the Vindolanda Wooden Writing Tablets: A Noninvasive Multianalytical Protocol for the Characterisation of Black Roman Inks
Source: J Anal Methods Chem. 2026 Feb 8;2026:5142007. doi: 10.1155/jamc/5142007 (PMC12884006; doi:10.1155/jamc/5142007)
Supplement: Supplementary file 1 — Supporting Information Additional supporting information can be found online in the Supporting Information section. [file JAMC-2026-5142007-s001.docx]

**Supporting Information**

**The making of the Vindolanda wooden writing tablets: a non-invasive multi-analytical protocol for the characterization of black Roman inks**

Giovanna Vasco^1,*^, Joanne Dyer^1,*^, Richard Hobbs^2^, Caroline Cartwright^1^

^1^ Department of Scientific Research, British Museum, Great Russell Street, WC1B 3DG, London, United Kingdom (UK)

^2^ Department of Britain, Europe and Prehistory, British Museum, Great Russell Street, WC1B 3DG, London, United Kingdom (UK)

*corresponding author: [giovanna.vasco@unisalento.it](mailto:giovanna.vasco@unisalento.it) ; [jdyer@britishmuseum.org](mailto:jdyer@britishmuseum.org)

Table S1 - Deconvoluted Raman parameters with the related standard deviations (Supplementary – G and D bands)

| ***Tablet BM reg. num.*** | | **FWHM_G_** | | **σ _FWHM G_** | **I _G_** | **σ _I G_** | **FWHM_D_** | **σ _FWHM D_** | **I _D_** | **σ _I D_** | **I _D_/I _G_** | **G band** | **σ _G band_** | **D band** | **σ _D band_** |
| --- | --- | --- | --- | --- | --- | --- | --- | --- | --- | --- | --- | --- | --- | --- | --- |
|  | ***69*** | 80.61 | 10.12 | | 0.29 | 0.02 | 194.60 | 28.87 | 0.30 | 0.05 | 0.87 | 1,594.04 | 1.95 | 1,360.60 | 1.13 |
|  | ***4*** | 62.68 | 0.85 | | 0.40 | 0.01 | 156.86 | 1.27 | 0.42 | 0.06 | 1.15 | 1,587.81 | 3.13 | 1,345.43 | 1.44 |
|  | ***9*** | 76.37 | 2.61 | | 0.23 | 0.07 | 152.51 | 18.72 | 0.23 | 0.09 | 1.23 | 1,594.07 | 1.81 | 1,358.72 | 4.77 |
|  | ***34*** | 82.84 | 5.98 | | 0.38 | 0.10 | 185.94 | 51.78 | 0.39 | 0.10 | 1.04 | 1,589.63 | 6.25 | 1,353.68 | 5.47 |
|  | ***63*** | 79.76 | 11.09 | | 116.45 | 163.93 | 226.72 | 38.77 | 97.74 | 137.48 | 0.84 | 1,589.04 | 3.41 | 1,360.42 | 1.57 |
|  | ***66*** | n.d. | n.d. | | n.d. | n.d. | n.d. | n.d. | n.d. | n.d. | 1.46 | n.d | n.d | n.d | n.d |
|  | ***71*** | 81.08 | 7.38 | | 0.36 | 0.11 | 219.45 | 35.26 | 0.35 | 0.07 | 1.35 | 1,589.72 | 4.20 | 1,352.70 | 5.32 |
|  | ***79*** | 65.45 | 4.16 | | 0.18 | 0.03 | 211.68 | 23.68 | 0.19 | 0.06 | 1.23 | 1,595.23 | 3.89 | 1,363.57 | 5.56 |
|  | ***128*** | 69.92 | 3.59 | | 0.17 | 0.02 | 218.32 | 28.55 | 0.18 | 0.02 | 0.98 | 1,594.42 | 4.59 | 1,364.99 | 2.34 |
|  | ***182*** | 75.88 | 4.61 | | 0.32 | 0.04 | 179.83 | 13.97 | 0.29 | 0.08 | 0.94 | 1,598.86 | 4.00 | 1,366.79 | 7.54 |
|  | ***22*** | 100.07 | 20.19 | | 0.34 | 0.11 | 175.82 | 19.00 | 0.28 | 0.10 | 0.85 | 1,586.83 | 5.71 | 1,361.24 | 4.03 |
|  | ***216*** | 66.91 | 6.95 | | 0.40 | 0.05 | 125.97 | 41.47 | 0.24 | 0.12 | 0.60 | 1,592.52 | 0.29 | 1,361.98 | 11.44 |
|  | ***223*** | 68.85 | 8.40 | | 0.27 | 0.05 | 265.19 | 16.58 | 0.27 | 0.05 | 0.87 | 1,591.87 | 2.15 | 1,353.28 | 2.44 |
|  | ***285*** | 69.62 | 3.39 | | 0.40 | 0.15 | 236.03 | 100.80 | 0.35 | 0.18 | 1.06 | 1,587.78 | 5.14 | 1,341.78 | 7.92 |
|  | ***301*** | 77.45 | 2.63 | | 0.31 | 0.05 | 288.20 | 105.89 | 0.28 | 0.05 | 0.88 | 1,595.07 | 1.81 | 1,356.26 | 11.11 |
|  | ***319*** | 85.40 | 7.83 | | 0.27 | 0.07 | 163.84 | 8.85 | 0.28 | 0.03 | 1.49 | 1,595.11 | 3.18 | 1,363.23 | 3.07 |
|  | ***320*** | 75.43 | 4.42 | | 0.26 | 0.08 | 308.43 | 77.67 | 0.29 | 0.08 | 1.02 | 1,597.90 | 1.74 | 1,350.55 | 8.49 |
|  | ***373*** | 67.22 | 5.47 | | 0.28 | 0.02 | 199.34 | 31.28 | 0.36 | 0.05 | 1.74 | 1,586.59 | 1.48 | 1,348.40 | 7.36 |
|  | ***399*** | 73.14 | 4.06 | | 0.37 | 0.01 | 161.97 | 21.66 | 0.37 | 0.15 | 1.45 | 1,591.36 | 1.13 | 1,350.93 | 6.39 |
|  | ***401*** | 95.48 | 19.24 | | 0.45 | 0.11 | 243.57 | 82.40 | 0.36 | 0.03 | 1.06 | 1,588.59 | 3.20 | 1,346.64 | 3.59 |
|  | ***427*** | 71.64 | 10.82 | | 0.31 | 0.09 | 251.80 | 40.67 | 0.23 | 0.07 | 0.62 | 1,593.66 | 2.47 | 1,357.74 | 4.58 |
|  | ***429*** | 69.36 | 7.51 | | 0.32 | 0.09 | 227.30 | 15.76 | 0.35 | 0.07 | 1.21 | 1,600.51 | 5.61 | 1,365.61 | 10.15 |
|  | ***114*** | 79.46 | 6.48 | | 0.16 | 0.05 | 172.49 | 37.12 | 0.17 | 0.07 | 0.71 | 1,596.48 | 1.04 | 1,358.62 | 3.16 |
|  | ***89*** | 72.30 | 3.49 | | 0.31 | 0.08 | 178.05 | 37.63 | 0.38 | 0.16 | 0.96 | 1,592.78 | 0.64 | 1,351.08 | 3.10 |
|  | ***12*** | 68.66 | 4.30 | | 0.40 | 0.04 | 188.11 | 43.98 | 0.46 | 0.10 | 0.87 | 1,593.95 | 0.09 | 1,351.34 | 3.46 |
|  | ***159*** | 78.21 | 4.92 | | 0.34 | 0.08 | 280.39 | 22.53 | 0.31 | 0.01 | 0.84 | 1,592.57 | 1.09 | 1,345.27 | 6.10 |
